# Creative problem solving and facial expressions: A stage based comparison

**DOI:** 10.1371/journal.pone.0269504

**Published:** 2022-06-22

**Authors:** Mritunjay Kumar, Satyaki Roy, Braj Bhushan, Ahmed Sameer

**Affiliations:** 1 Department of Design, Indian Institute of Technology Kanpur, Kanpur, Uttar Pradesh, India; 2 Department of Humanities and Social Sciences, Indian Institute of Technology Kanpur, Kanpur, Uttar Pradesh, India; 3 Department of Humanities and Social Sciences, Indian Institute of Technology (ISM) Dhanbad, Dhanbad, Jharkhand, India; Valahia University of Targoviste: Universitatea Valahia din Targoviste, ROMANIA

## Abstract

A wealth of research indicates that emotions play an instrumental role in creative problem-solving. However, most of these studies have relied primarily on diary studies and self-report scales when measuring emotions during the creative processes. There has been a need to capture in-the-moment emotional experiences of individuals during the creative process using an automated emotion recognition tool. The experiment in this study examined the process-related difference between the creative problem solving (CPS) and simple problem solving (SPS) processes using protocol analysis and Markov’s chains. Further, this experiment introduced a novel method for measuring in-the-moment emotional experiences of individuals during the CPS and SPS processes using facial expressions and machine learning algorithms. The experiment described in this study employed 64 participants to solve different tasks while wearing camera-mounted headgear. Using retrospective analysis, the participants verbally reported their thoughts using video-stimulated recall. Our results indicate differences in the cognitive efforts spent at different stages during the CPS and SPS processes. We also found that most of the creative stages were associated with ambivalent emotions whereas the stage of block was associated with negative emotions.

## Introduction

The cognitive mechanisms underlying the creative process have been the focus of creativity research for decades [1]. Numerous sources of information, knowledge, skills, and emotions are utilized in different ways during the creative process [2]. Therefore, understanding how individuals take different approaches and learn to be creative becomes crucial to support their creative development.

Recent years have seen a rise in the number of studies in understanding the creative subprocesses and stages [3–5]. While most of the studies have focused on understanding the granularity of the creative process, it is essential to identify the creative act in its totality [6]. It is crucial that we evaluate the similarities and differences between the creative and non-creative acts to develop a holistic understanding [2]. Nevertheless, this area has received scant attention, and no direct study has empirically investigated the nature of the either processes [7–9].

It is no surprise that a significant part of the creative process is emotional [10], from the initial decision to try something new to the skills for maintaining enthusiasm and perseverance for creative endeavors [8]. Unfortunately, assessing emotions during the creative process remains a challenge due to methodological complexities. Methods such as experience sampling methods and diary studies have successfully captured the dynamics, but they do not account for the in-the-moment emotional experiences during the creative process. There is a strong need for a novel approach to capturing emotions in real-time during the creative process.

This article is exploratory in nature and examines the process-related differences between the CPS and SPS processes, as well as captures the in-the-moment emotional experience of the individuals during the CPS process. The specific questions addressed in this article include:

RQ1: What stages of the CPS and SPS require the greatest amount of cognitive effort from the individuals?
RQ2: What types of emotions are experienced during the various stages of the creative process?

### Background of the study

#### The dynamics of the creative process

The connotation of the word ‘dynamic’ supports the concept of the creative process not being linear [2] p. 295 explained the creative process as “a succession of thoughts and actions that leads to novel and adapted productions.” Research into the creative process has progressed rapidly since [11] four-stage model of creativity and several models have emerged over the past few years (for a detailed overview, see [2]) despite the absence of consensus on the definition of the stages. Cropley and Cropley [12] have mentioned seven stages—preparation, activation, generation, illumination, verification, communication, and validation. In Wallas’ four-stage model, Sadler-Smith [13] proposes a five-stage model, with an intimation phase added between incubation and illumination. Sawyer’s emergent model [14] examined six subprocesses (intuition, idea emergence, iteration, experimentation, and exploration). More recent work by [3] examined the concurrent sequence between the subprocess of generation, selection, exploration, evaluation, refinement, comparison, synthesis, and application. Other researchers have explored the stages of problem recognition, idea generation, idea evaluation, and solution validation in creative problem solving [15]. Furthermore, for an understanding of the creative act in its entirety, it is necessary to identify similarities and differences between the processes that result in creative outcomes and those that result in noncreative outcomes [2, 6]. Lubart presented four hypotheses as possible explanations for the differences. Under the first approach, creativity and non-creativity can be viewed as separate constructs that lead to creative and non-creative outcomes. The second hypothesis doesn’t differentiate these two processes and considers a creative and noncreative process continuum. Third, depending on the quality of knowledge used, the same process can lead to highly creative, moderately creative, or non-creative outcomes. Lastly, these processes may entail the same stages and may also involve the same amount of time spent at each stage, with the only variation being the quality of execution at each stage. Mumford and colleagues also outlined four hypotheses regarding the differences between creative and regular problem-solving processes. First, creative problem solving (CPS) involves ill-defined problems, as opposed to regular problem-solving. Second, CPS allows higher degrees of divergent and convergent thinking wherein routine tasks permit applying previously known procedures and information to solve the problem. Third, the difference lies in the multiple cycles of divergent and convergent thinking involved in CPS instead of regular problem-solving. Finally, the CPS process involves reorganizing and restructuring the information, while the routine, non-creative process just recalls the information based on the existing knowledge. These differences, though, require further investigation as there is no empirical evidence that can be used as a comparison between CPS and the simple problem solving (SPS) processes.

#### Emotions and the creative process

Similarly, understanding the emotional processes that contribute to creativity becomes essential as they influence higher-level cognitive functions during the creative process, such as perception of the stimuli, judgment, decision making, and reasoning [16, 17]. Phenomenology research into creativity and emotions shows a wide range of emotions occurring during the creative process across many domains. Several creative individuals, e.g., artists, musicians, scientists, designers, describe the feelings of mixed emotions such as joy, happiness, and pain during the long processes of working and reworking on the creative problem to realize the idea of the product or outcome [18, 19]. Some long-standing views have examined the role of positive emotions in enhancing cognitive flexibility and creativity [8]. Others have explored the relationship between negative affects compared to neutral mood states in promoting creativity by helping individuals be more focused, critical, and determined in producing a creative outcome [20, 21]. The third paradigm produced reliable evidence for ambivalent emotions association with creativity [22], where highly activated positive (excited) and negative (angry) states were linked to high creative engagement and deactivated positive (relaxed) and negative (discouraged) states were linked to lower creative engagement [23].

Notably, a few studies have also investigated the dynamics of emotions for different creative stages. Peilloux and Botella [24] for example, found that the creative stages of immersion, thinking, research, inspiration, and insight were associated with positive emotions and the stages of judgment, experimentation, and planning were associated with negative emotions. Most recently, Kumar et al. [25] examined the dynamics of emotions using the eight design subprocesses, where positive affects dominated the conceptual phase of the design process whereas negative affects dominated the embodiment phase of the design process.

#### Existing measures of the creative process

*Protocol analysis*. Protocol analysis has emerged as an important tool for behavior analysis during the dynamics of the creative process [3, 5, 26, 27]. Concurrent verbal protocols or the think-aloud method involves participants verbalizing their thought processes while performing the task in real-time using their short-term memory [28]. In the retrospective protocol method, participants retrieve the information of the already completed task that has been stored in their memory. Both these methods have their own advantages and limitations. Gero and Tang [29] have emphasized that both these methodologies achieve similar results with respect to examining the process-oriented aspects of the creative design process.

*Markov’s chain analysis*. Recent years have seen a rise in utilizing the Markov’s chain analysis to study the creative subprocess sequence. A Markov Chain is a stochastic model for predicting, estimating, or guessing the result of an event based on the preceding state and its action [30–32]. Pringle and Sowden [5] compared shifts of the associative mode of thinking with the analytical mode of thinking during the processing of emotional input. Moreover, Kan and Gero [33] examined design protocols from the standpoint of the sequential order of the Function Behavior-Structure (FBS) processes using the first-order Markov chain for various design processes. Nevertheless, the Markov analysis can be performed on any coding scheme. Ergodic Markov chains have the property of achieving a single stationary state distribution as time progresses. Generally, it is represented as a row vector π whose entries are probabilities that add to 1, and given the transition matrix P, it satisfies

πP=π (1)

where π is referred to as the equilibrium distribution of a chain. Computing a stationary state distribution makes it easy to statistically compare different distribution probabilities.

#### Existing measures of emotions during the creative process

Most of the published research in measuring creativity and emotions together has relied primarily on diary studies and experience sampling methods [34, 35]. Even though these methods offer greater ecological validity, they are costly, time consuming, and do not account for in-the-moment emotional experiences of the creators. A further step in this direction should include the use of various technological advances that enable the investigation of the physiological and behavioral measures of the creative process and emotions [36]. A recent study employed eight body postures to decode an individual’s emotions during the creative design process using Kinect and a machine learning classifier [37]. Several articles have also asserted that facial expressions can be used to study emotions during the creative process [17, 38]. Maybe the concept of measuring facial Action Units (AUs) can be utilized to map the corresponding emotions that arise at different stages of the creative process.

### Present study

#### The research gap

Several articles have asserted that facial expressions can be leveraged to study emotions of the individuals during the creative process. To the best of our knowledge, no study has examined the dynamics of emotions during the CPS process using facial expression data.

Consequently, the two primary goals of this study are to examine the process related difference between the CPS and SPS processes and to capture in-the-moment emotional experiences of individuals using facial expressions during the CPS process.

#### Objective of this study

More specifically, the objectives of this study are -

1. To compare the CPS and SPS processes using Protocol analysis and Markov chains.
2. To classify different stages of CPS process based on automatic facial AU data combinations.

## Method

### Participants

A total of 69 participants volunteered for the study, of which two opted out of the study, citing discomfort in wearing the headgear during the experiment, while the data of three participants were excluded due to the wrong orientation of the facial camera during the experiment. Thus, the study was carried out on a convenience sample of 64 adults randomly assigned to the CPS and SPS task groups. The CPS and SPS groups comprised 33 (17 men and 16 women) and 31 (18 men and 13 women) participants, respectively. The mean age of CPS group was 25.24 years (Men 25.59 years, SD 1.06; Women 24.88, SD 0.96), while the mean age of SPS group was 25.45 years (Men 25.78, SD 0.81; Women 25.00, SD 0.71). All the participants were postgraduate students ranging from several disciplines: mechanical engineering, electrical engineering, fine arts, design, civil engineering, psychology, literature, philosophy, and economics. The presence of glasses, beards, and mustaches were the exclusion criteria. The participants were recruited through an advertisement and were monetarily compensated. They were briefed about the study, and their participation was confirmed after they signed the informed consent form. The study protocol (IITK/IEC/2018-19/II/7) was approved by the Institute Ethics Committee of the Indian Institute of Technology Kanpur.

### Materials

#### Tasks

**Fig 1:**
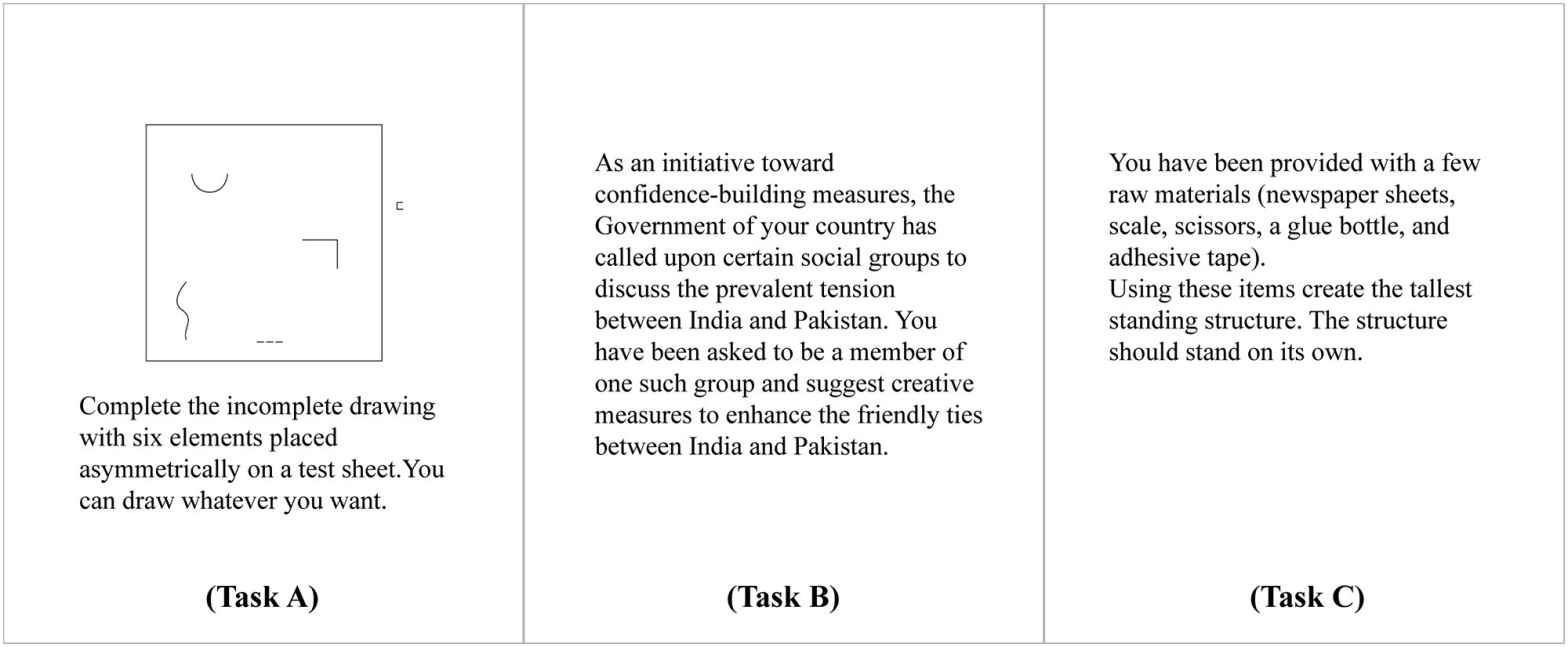
CPS group tasks.

**Fig 2:**
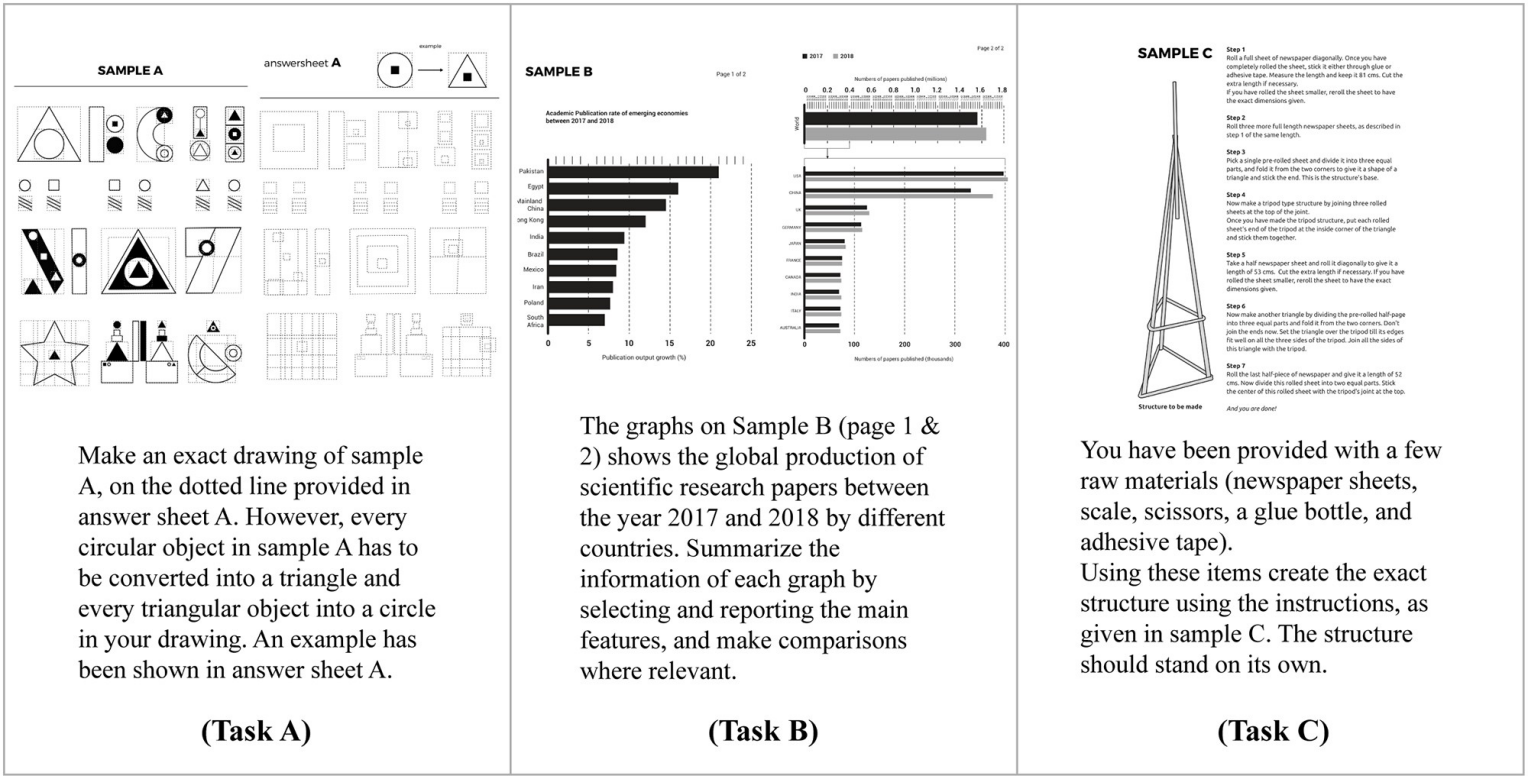
SPS group tasks.

Two different sets of tasks, CPS and SPS, each comprising of three different activities, were used in this study. The CPS task consisted of three different tasks (Fig 1), a drawing task [39], a writing task [40], and a structure making task [41]. The diversity of these creativity tasks allowed more room for creative engagement. They were chosen owing to the fact that working on a variety of creative tasks and switching between them enhances divergent and convergent thinking by reducing cognitive fixation [42]. The SPS task consisted of a drawing task, a writing task, and a structure-making task that were tailored to demand the same amount of effort as solving the creative tasks (Fig 2). The difficulty levels of the two sets of tasks were assessed beforehand by eight judges who rated the creative components (1 = not at all creative, 5 = very creative) and difficulty levels (1 = very easy, 5 = very difficult) of the tasks on a 5-point Likert scale. The two tasks did not differ in terms of their difficulty levels (t = −0.323, df = 14, p = 0.751). In contrast, the CPS tasks were rated significantly higher on creativity as compared to the SPS tasks (t = 15.82, df = 14, p < .001).

#### Capturing the facial expressions

**Fig 3:**
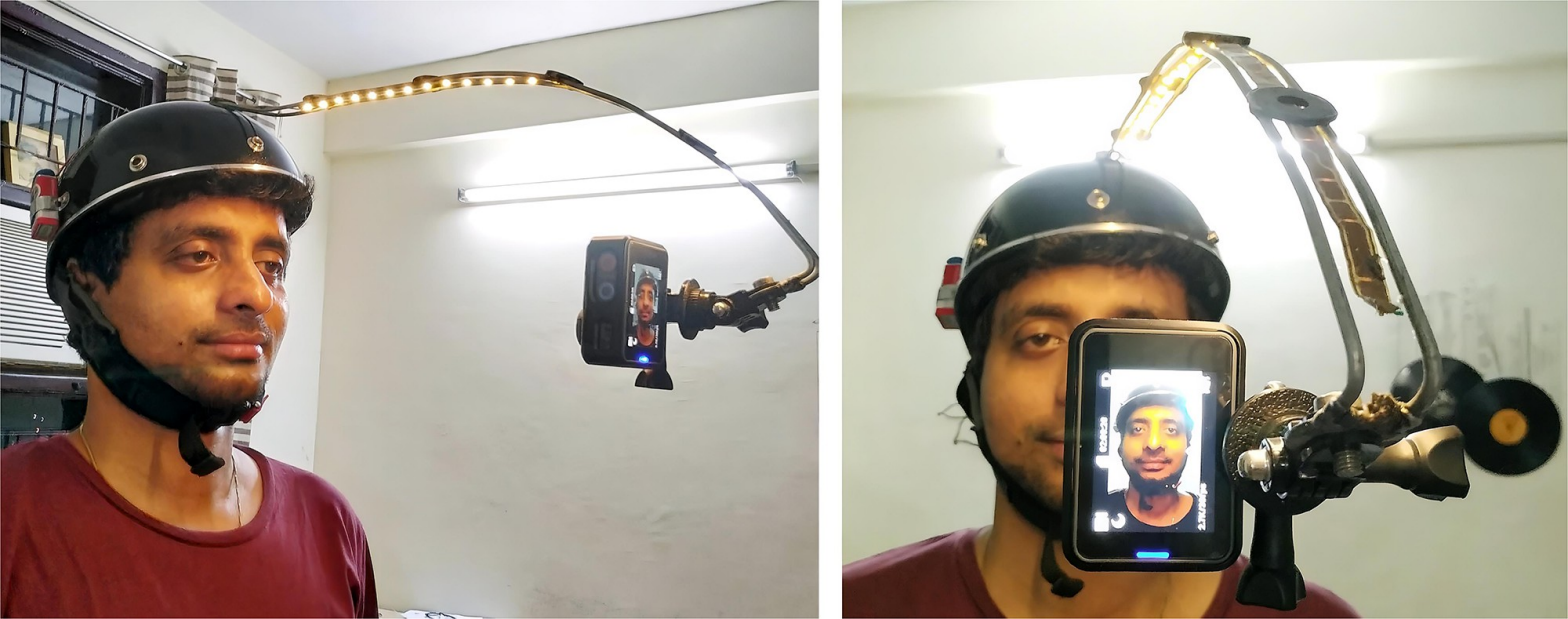
Facial profiles of the participant with headgear set up. The left figure presents the 3/4th view of the face, while the right figure presents the frontal profile. In both instances, the face has been appropriately illuminated using LEDs.

Participants’ facial expressions were captured using a custom-made head-worn camera. The aim was to ensure that the frontal faceview was always in view of the camera, regardless of the participant’s head movements during the task. An AKASO V50 pro sports camera was mounted on the headgear facing the participant at an approximate distance of 12.25 inches from their nose tip. The face was also illuminated by a strip of LED lights embedded in the headgear, as shown in Fig 3. The participants’ facial expressions were recorded while solving the tasks for 30 minutes at the rate of 25 frames per second in 720p.

A manipulation check was performed to ascertain that the headgear did not interfere with the task performance by administering the same to 18 randomly selected matched participants who were not part of the main study. Nine participants were required to wear the headgear, while the remaining nine did not and were asked to complete the alternative usage test [43] where they were asked to provide ‘five different usages of a brick’ within two minutes. The z-scores indicated no difference between those wearing the headgear and those who did not on fluency (z = −0.97, p = 0.39), flexibility (z = −0.225, p = 0.87) and originality scores (z = −0.94, p = 0.39).

### Procedure

**Fig 4:**
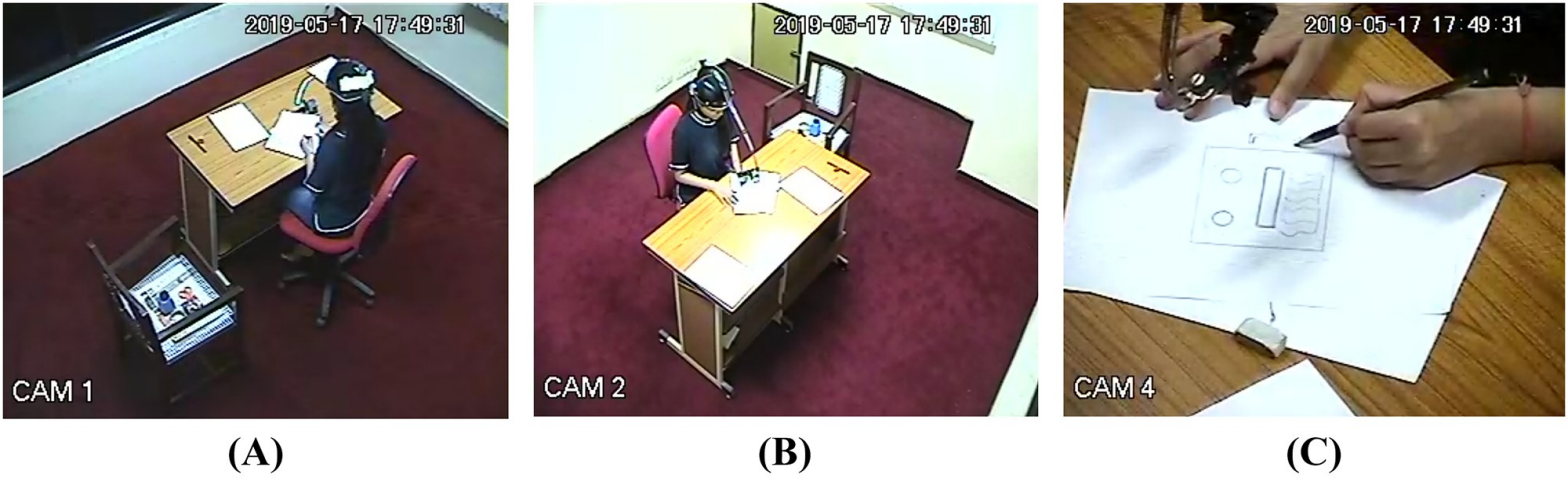
A three-camera view of the participant’s activity during the process. (A) A close-up view of the output is captured by the CAM4, (B) a medium close-up 3/4th view is captured by the CAM2, (C) and a zoom-out view of the overall activity is captured by the CAM1, in the psychology lab.

The experiment was conducted in the one-way glass room of the psychology lab at IIT Kanpur. This room was equipped with three dome cameras to record all the activities of the participants. Besides observing the participant through the one-way glass, the experimenter could observe the session from the observation room by controlling the camera movements using a control panel. The three cameras captured the whole process providing a close-up view, a 3/4 profile, and a backside profile (see Fig 4). The one-way glass room had only a working table, and a chair in addition to a table meant for the raw materials. After establishing the rapport, the participants were provided with the informed consent form upon reaching the venue. Upon signing it, they were instructed to wear the headgear and continue the interaction with the experimenter. This was done in order to make them accustomed to the headgear. As soon as the participant approved of working for 30 minutes on the tasks while wearing the headgear, they were instructed to proceed to the one-way glass room. The instructions were read aloud to them, and thereafter the CPS or SPS set was provided to them. The participants could start with any task and switch between tasks or work simultaneously on different problems. At this point, the participants were instructed not to read the three tasks until they heard the 1st buzzer. Doing so ensured that we did not lose out on any possible observation of the spontaneous occurrence of stages just after reading the task/tasks. The participants began working on the tasks following the first buzzer, and their sessions were recorded. While the dome cameras recorded the entire session, the head-mounted camera made separate recordings of the facial expressions. The participants were instructed not to think aloud to avoid any manipulation of the facial AUs. Participants were also periodically reminded of the time passed every 10 minutes through the buzzer sound. The total time for completion of all three tasks was 30 minutes. The task activity was followed by a short break of five minutes. Following the short break, the participants were asked to describe their thinking process during the activity based on some questions and instructions. Using the video stimulated recall, participants watched the whole video recording of their activity, where they were asked to reflect retrospectively on the process, speak aloud, and report their experiences. They were free to pause the video and speak about the process. This session was audiotaped. The total time to administer the whole experimental procedure, including the retrospective recall, was approximately 110 minutes per participant.

### Data analysis

#### Segmenting the protocols

The video of facial expressions was synchronized with the timeline of the three dome camera recordings from the beginning to the end of the activity and the audio recordings with the video timelines. Adobe Premiere Pro CC was used to trim and synchronize videos and audios using the frame-matching features. Audio MP3 for each participant was mastered and boosted for better sound quality. This was followed by transcription of the verbal utterances on a word file. The transcribed verbal utterances were further divided into small units/segments/moves based on one main verb per parse [26] along with the timestamps in the datasheet for each participant. The start and end of a verbal utterance were timestamped based on the time interval of the recorded video that the participants were referring to. After parsing the verbal utterances, video recording observations that were not reported as verbs or actions during the retrospective verbal utterance process were separately segmented and were added to the same excel sheets with timestamps. They can be categorized as task-related actions (e.g., reading the task description, sketching, making the prototype, arranging the raw materials, etc.) and other actions (e.g., standing up, relaxing the body, etc.). The segmentation of actions based on video observation became more robust by capturing details that were obscured by verbal utterances alone.

#### Coding the segments

Following the review of the segmented protocols, seven categories, namely preparation, ideation, illumination, evaluation, verification, production, block, or other were developed as coding scheme with the attributes of the different modes of thinking based on the literature review. Each segment was assigned to one category only. Table 1 summarizes the coding scheme based on which a total of 18,355 segments were coded for all the 64 participants.

**Table 1:** Details of information category, example segments, and code explanation from participants’ protocols, all coded into seven stages.

Information Category	Protocol examples taken from the CPS and SPS groups	Code Category	Code Explanation
Task-related action	Reads the Instruction sheet and the question paper	Preparation	One prepares to solve the problem by acquiring information, searching for answers, setting the problem, and find the solution approach [11, 12]
Task-related action	Picks up the raw materials from the chair		
Verbal Utterance	“I needed some time to think on this topic”		
Verbal Utterance	“I saw this hut as going into that shape	Ideation	It’s a generative process that includes the mental construction of various types of pre-inventive structures, that can become potential novel solutions [3, 44, 45]
Verbal Utterance	“you know this curve is like I felt that about making a violin”		
Verbal Utterance	“so the first thing that to my mind was a kettle”		
Verbal Utterance	“and while folding this sheet I suddenly came up with the 2nd task ka final answer here”	Illumination	It’s the sudden appearance of an idea with immediate certainty [46, 47]
Verbal Utterance	“this came very suddenly to me”		
Task-related action	picks up another sheet and makes the cone	Production	Production corresponds to the elaboration and transformation of an idea into a physical entity through action [48, 49]
Task-related action	Draws the 2nd element’s rectangle		
Task-related action	shades the 2nd element’s lower right triangle		
Verbal Utterance	“This base was somehow able to hold the structure still”	Evaluation	During this stage, the individual assesses the idea and the produced output [50, 51]
Verbal Utterance	“Here I made it by mistake because this part confused me”		
Verbal Utterance	“the sheet was very thin that is why my structure was falling down		
Task-related action	redraws the eye	Refinement	It is the process, the creative goals or work is revisited, refined and reformulated [3, 52]
Verbal Utterance	“So I redrew this part again to match this section”		
Verbal Utterance	“I cut this part because it was longer than 81 cms”		
Verbal Utterance	“here I was confused about making the star shape”	Block	Creative block is an inability to work due to external or internal factors [45, 53]
Verbal Utterance	“but I was not sure what to make”		
Verbal Utterance	“After much thinking, nothing was coming to my mind for this task”		
Verbal Utterance	“I have never done such a task before	Other	Utterances or moments that were not related to the task.
Verbal Utterance	“Haha, I don’t know why I did that”		

When dealing with a large number of categories, coding requires a high level of reliability. Accordingly, after the lead author completed this exercise, an independent coder who was blind to the experiment but was proficient in coding schemes and creativity research coded 1476 segments randomly chosen from the CPS and SPS groups. Cohen’s kappa was computed for these 1476 segments to determine intercoder reliability. We observed strong agreement in 82% of the segments (k = .816, p < .001) after correction for chance. In the event of disagreements between the coders, the coding categories were discussed between the two coders in order to categorize the protocols into different stages based upon different modes of thinking. Upon resolution of the discrepancy between the coders, the codes were adjusted to be consistent across all protocols for each participant.

#### Markov’s analysis to study the transition between different stages

Based on the coding for the segments in the excel sheet, we calculated the probability of transition from one state to the next for each of the eight categories, providing us with an 8 by 8 matrix for each participant. Using these probability matrices, a stationary state distribution matrix with one row and eight columns was created using R studio for each participant to represent the differences in cognitive effort exerted at different stages of the task solving process. The analysis of Markov’s stationary state distribution in this study was based on the work by Kan and Gero [33].

#### Transition state diagrams

The transition diagrams provide a visual representation of the transition of events with their probabilities. Based on the analysis of Jeong’s work [54], the transition state from one stage to another, for each participant, the sum of these frequencies was taken, and a relative frequency was computed for each transition. This provided us with a single transition matrix containing 64 probabilities of the transitions for the CPS and SPS groups, respectively.

In these transition diagrams, the creative stages are represented by the nodes that are linked to the other nodes using directional arrows. These directional arrows represent the relative frequency from one stage to another and the width of the arrow represents the strength of the transitional probability. Numbers in the transitional state diagrams represent the probability of one stage being followed by another stage.

#### Facial expression analysis

*Automatic facial action coding system*. Facial Action Coding System [55] is a rigorous and psychometrically robust system that utilizes facial muscle movements to represent them as action units (AUs) to describe facial activity. Emotion-specific AUs or the combination of AUs can be found in EMFACS [Ekman & Friesen, 1983 [Unpublished]]. Several researchers across different domains have used automatic facial expression recognition software to characterize a specific set of emotions [56].

This study leveraged the OpenFace 2.0 [57] program to extract the facial action units from the synchronized videos of all the sessions. Based on comparing the performance of automatic facial expression recognition tools, OpenFace has been shown to be superior to the high-paid commercial softwares, e.g., Nodlus FaceReader, and Affectiva in detecting automatic facials. AUs with better accuracies [58]. Another advantage of OpenFace is that it is available in the open-source domain (https://github.com/TadasBaltrusaitis/OpenFace). This program is trained on different facial datasets and uses a linear kernel support vector machine to recognize individual AUs. The system recognizes seventeen specific AUs (1, 2, 4, 5, 6, 7, 9, 10, 12, 14, 15, 17, 20, 23, 25, 26, and 45). This study used only 12 AUs and their combinations that correspond to seven emotions. Details regarding the combination of AUs related to specific emotions used in this study can be found in [Ekman & Friesen, 1983 [Unpublished], 59, 60].

**Fig 5:**
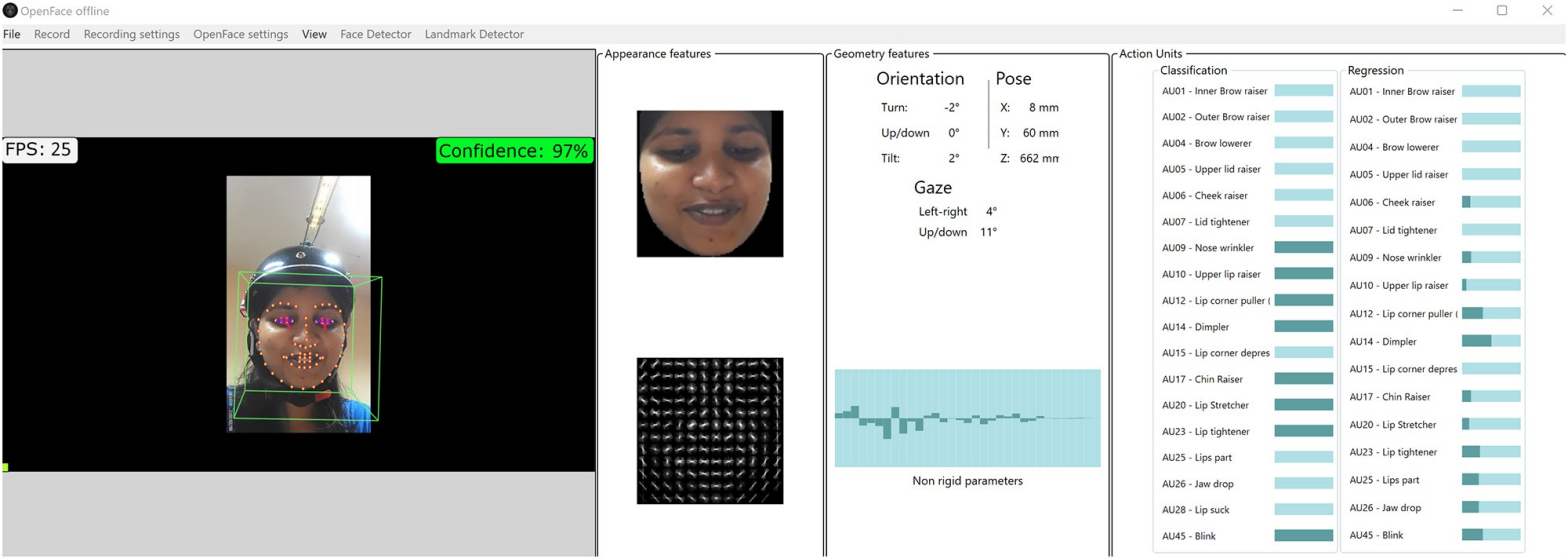
User interface of the OpenFace 2.0 program. Based on video and image outputs, this program detects and extracts participant facial features such as facial landmarks, head pose, eye gaze, and facial Aus.

For each participant, OpenFace generated a total of 45000 frames based on the video duration of 30 minutes. These 45000 frames (rows) contained continuous AU data from 0 to 5 (intensity of AUs) for 12 facial AUs, along with the timestamp and confidence level. In most cases, AUs is regressed with high levels of confidence, usually 98%. To increase internal validity, frames with confidence levels less than 95% were removed from the analysis. Fig 5 demonstrates the OpenFace graphical user interface while simultaneously collecting the variables in a spreadsheet.

#### Support vector machine to classify the creative stages based on facial AU combinations

The dataset generated by OpenFace required further processing before classification, as the facial AU datasets were highly imbalanced (typically a result of an unequal distribution of classes within a dataset) with some AUs with 0 values indicating no movement of specific muscles, while other AUs displayed continuous values (above 0), indicating movement intensity of the muscles at different time intervals.

As part of the feature set in this study, seven different combinations of AUs are used to represent seven different emotions to predict the target class, comprising of six stages. During the data analysis, the datasets of ‘illumination’ and ‘others’ stage were discarded as ‘illumination’ occurs within a fraction of a second [61] and is, therefore, inaccessible by retrospective protocol analysis and ‘others’ did not contain tasks-related information. Further, by eliminating these stages, the model’s performance improved by reducing the data imbalance issue. The final dataset for the CPS and SPS groups used for the SVM classification is presented in Table 2.

**Table 2:** CPS and SPS datasets of labelled in six stages (rows) and 12 AUs (columns) for all participants, used for the SVM classification.

CPS Dataset	SPS Dataset	Stages
307599 rows * 12 Columns	631997 rows * 12 Columns	Preparation
319334 rows * 12 Columns	3820 rows * 12 Columns	Ideation
47475 rows * 12 Columns	61185 rows * 12 Columns	Evaluation
127176 rows * 12 Columns	75230 rows * 12 Columns	Refinement
531279 rows * 12 Columns	554815 rows * 12 Columns	Production
2947 rows * 12 Columns	4109 rows * 12 Columns	Block

Before the model generation in SVM, we used a random undersampling approach to balance our facial AUs dataset using python libraries. We leveraged the imblearn.undersampling_sampling package in python programming language to undersample the data and generate a new dataset. The RandomUnderSampler function results in random selections of examples from the majority class. The parameter ‘replacement’ is set to false to prevent the repetition of the same example that requires deletion from the training dataset. This is followed using the fit_resample function to under sample the dataset and thereafter, train_test_split function has been leveraged to split into the training and testing dataset. 80% of the data were split into training sets and 20% as test sets.

We used a polynomial kernel function with degree 4 using Python to classify the stages, as other kernels did not provide high accuracy in predicting the stages based on different combination of AUs. Beyond this degree, the model became saturated. Moreover, our SVM model utilized the C parameter to avoid misclassification of the training data. Decision function shape is used as an ovo (OnevsOne) classifier to serve as a binary classifier for all pairs of classes, as well as the training utilizes the fit function to obtain a fitting of the model to the input training instances while testing utilizes the predict function to make predictions about the testing instances.

## Results and discussion

### Comparing the distribution of stages during the SPS and CPS processes

Since the computed stationary state distributions of the stages were not normally distributed, the Mann Whitney U test was conducted. The results of the statistical analyses are summarized in Tables 3 and 4.

**Table 3:** Mean values of the stationary state distribution of the creative stages between the CPS and SPS groups.

Creative Stages Distribution	CPS group (N = 33) Mean (SD)	SPS Group (N = 31) Mean (SD)
Preparation	0.21 (0.05)	0.53 (0.04)
Block	0.02 (0.06)	0.01 (0.01)
Ideation	0.37 (0.10)	0.01 (0.02)
Evaluation	0.09 (0.03)	0.08 (0.03)
Refinement	0.07 (0.03)	0.04 (0.02)
Production	0.23 (0.04)	0.32 (0.03)
Other	0.01 (0.01)	0.01 (0.01)
Illumination	0.00 (0.00)	0.00 (0.00)

**Table 4:** Results of the Mann Whitney U test comparing the stationary state distribution of stages between the CPS and SPS groups.

Creative Stages Distribution	z	p
Preparation	-6.878	0.000***
Block	-0.886	0.375
Ideation	-6.572	0.000***
Evaluation	-1.432	0.152
Refinement	-3.437	0.001***
Production	-5.914	0.000***
Other	-1.192	0.233
Illumination	-2.696	0.007***

Statistical analysis revealed a significant difference in the distribution of stages during the CPS and SPS processes. Participants in the CPS group predominantly spent their cognitive efforts towards the stages of Ideation (z = −6.572, p < 0.05), Illumination (z = −2.696, p < 0.05), and Refinement (z = −3.437, p < 0.05).

Task A’s ideation phase involved participants generating pre-inventive structures from incomplete elements provided to them, which encouraged them to diverge and consider alternate solutions. For example, Participant 01 or P01 (CPS) commented, *“This curvy shape looked like a girl and a vase”* whereas, P02 (CPS) associated the shape with surrealism and reported, “*It looked like a Dali painting*.*”* The majority of participants for Task B expressed ideas based on the combination of the previously stored schema for having common festivals as interaction points between two countries that could facilitate cross-cultural exchanges. For example, P18 (CPS) reported, *“we can have movies like Bajrangi Bhaijaan which will bring more unity among each other”* while P12 (CPS) reported, *“then I thought about the food as a medium to connect these two countries*.*”* During Task C, most participants were interested in creating a strong and stable foundation for the structure. For example, P28 (CPS) reported, *“to fir I got the idea about making a strong base that will hold the tower*,*”* and P07 (CPS) reported, *"I will have to make many legs where this long heightened thing can stand*.*”*

In the illumination stage, some of the participants credited the emergence of insight to random associations during idea generation that somehow emerged spontaneously as an appropriate solution. For example, P02 (CPS) during Task A reported, *“you know when I saw this*, *I was thinking of many things*, *and immediately this idea suddenly came*.*”* Some participants attributed the spontaneous occurrence of insights to chance; for example, P30 (CPS) during Task A reported, *“I mean what a coincidence that I bought a chair today and when I saw this it clicked here*.*”* During Task C, P06 (CPS) reported, *“and then here the idea came to me just like that*.*”* It is worth noting that time constraints played a crucial role in some participants’ sudden appearance of insights. For example, P07 (CPS) reported during Task C, *“and I am like very capable if the time is limited*, *like here where I came up with the leg spread concept*.*”*

A substantial number of enhancements were made during the refinement stage to produce a better output. Many participants returned to the previous tasks and refined their work as they worked on the current task. Since they were occupied with other tasks, they were more likely to incubate, evaluate their previous responses, and then make changes.

Participants in the SPS group spent majority of their cognitive efforts during the Preparation (z = −6.878, p < 0.05) and the Production stage (z = −5.914, p < 0.05). The preparation stage required a considerable amount of concentration and attention. Task A demanded considerable focus on the deliberation of mentally converting the shapes. For example, P06 (SPS) reported, *“these shapes were simple but required a lot of attention as you need to think the reverse of it”* while P12 (SPS) reported, *“I became habituated with the drawings and the conversion thing*.*”* During the preparation stage of Task B, participants worked on gathering the information from the graphs, translating the data, and arriving at relevant conclusions. For example, P15 (SPS) reported *“these graphs were simple but I was reading them again and again to find the connection”*. Similarly, during Task C, P13 (SPS) reported, *“so I read these instructions one by one and it required focus*.*”*

During the production stage, participants spent their cognitive efforts in creating or transforming their ideas or thoughts into a tangible solution. In task A, for example, participants focused most of their efforts on implementing their ideas through sketching on the grid answer sheet. We observed an average increase in participants’ drawing speed after the 6th to 7th element. As participants became familiar with the grid and the reference sheet, their drawing speed increased. During the task B production stage, participants spent their efforts in writing their conclusions after making sense of the data. During Task C, participants tried to develop the structure prototype based on the instructions provided. It is interesting to note that some participants spent considerable time unraveling the adhesive tape, while others cut multiple strands of tape and stuck them to the table for convenience and time savings.

### Stage transition diagrams between different stages

**Fig 6:**
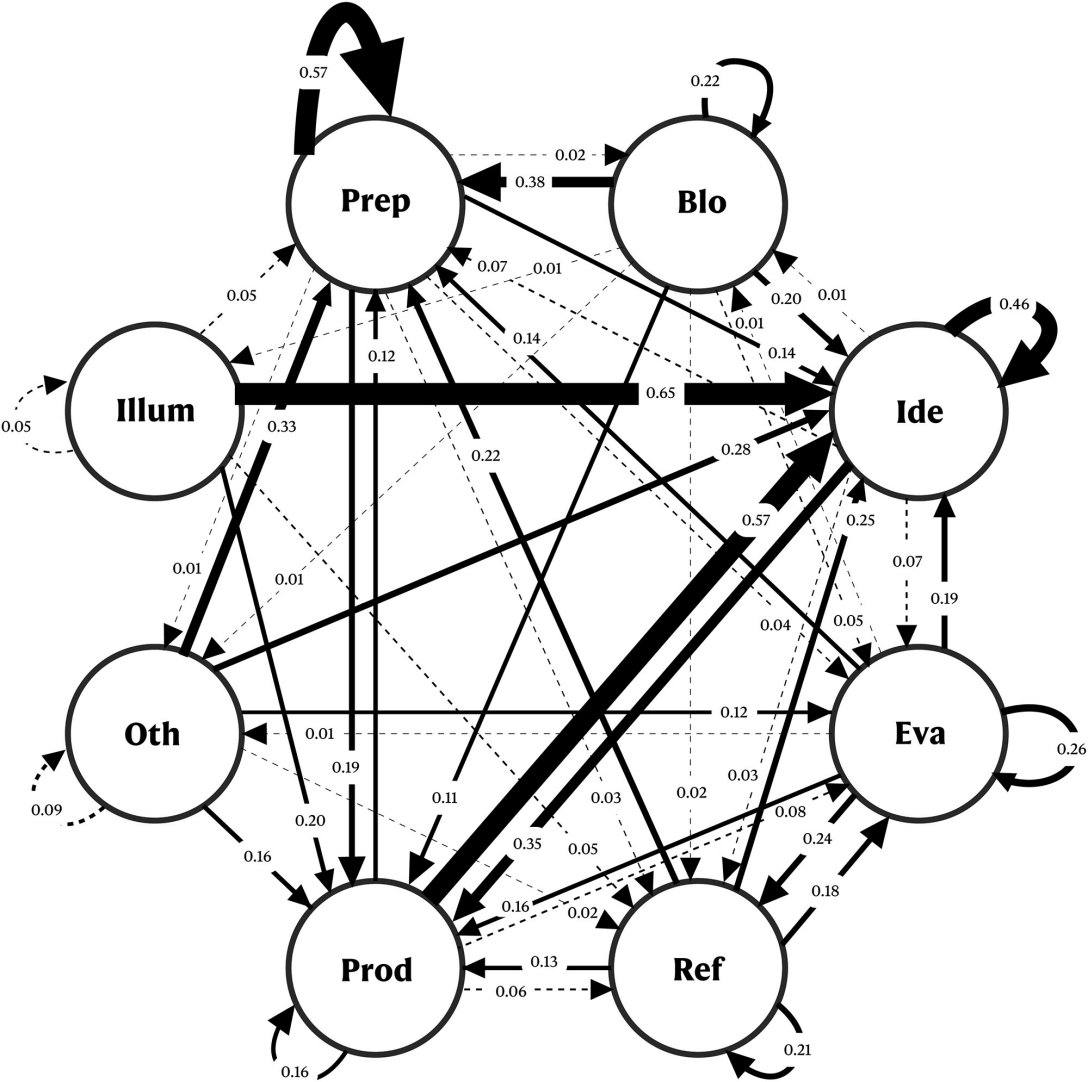
Diagram of transitional states for the various stages of the SPS group. Blo = Block; Ide = Ideation; Eva = Evaluation; Ref = Refinement; Prod = Production; Oth = Others; Illum = Illumination.

**Fig 7:**
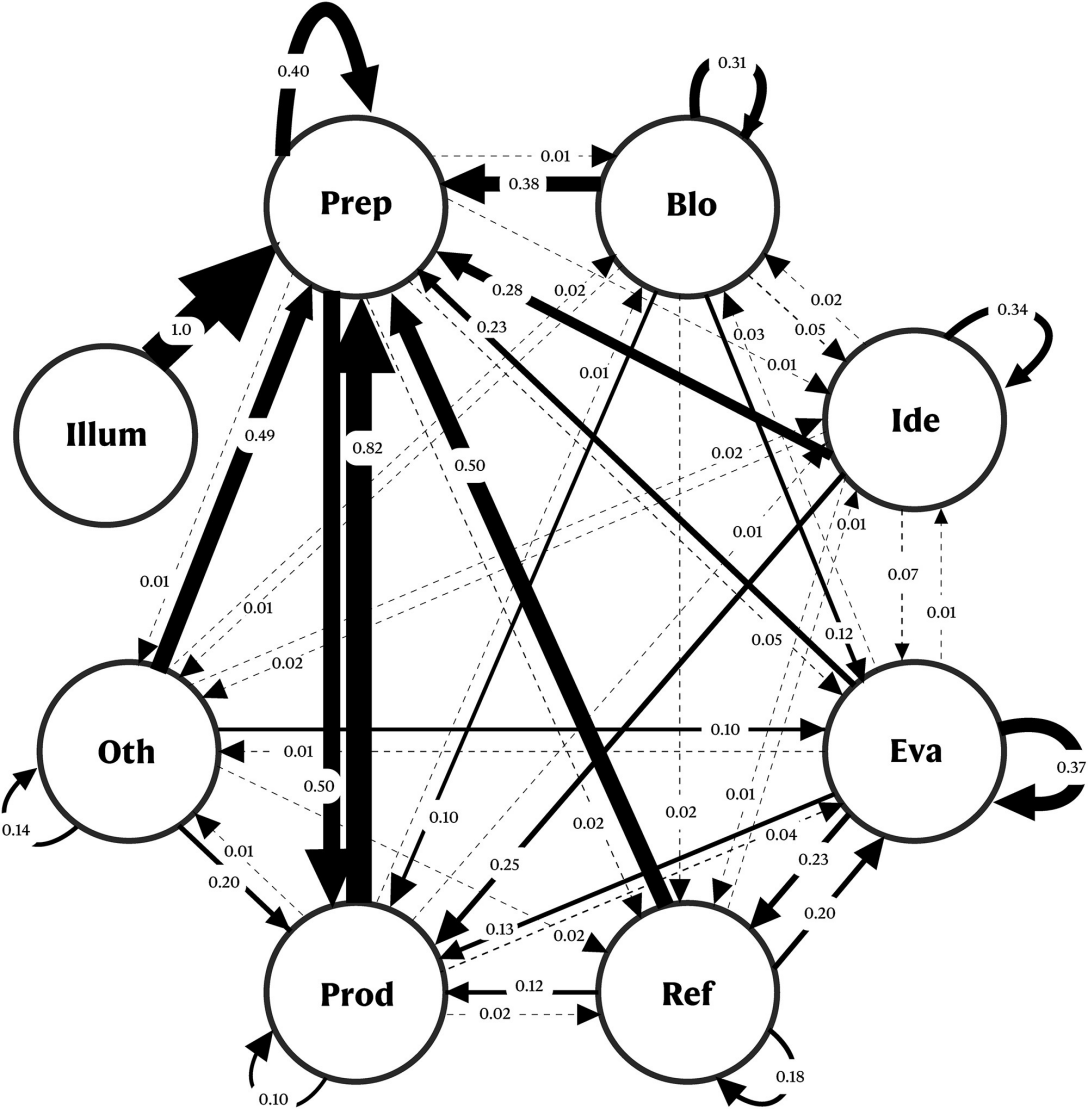
Diagram of transitional states for the various stages of the CPS group. Blo = Block; Ide = Ideation; Eva = Evaluation; Ref = Refinement; Prod = Production; Oth = Others; Illum = Illumination.

As illustrated in Figs 6 and 7, the participants of CPS group expressed more thought sequence from the production to the ideation stage (57%) than the SPS group (1%). As participants developed the idea they produced them, and then immediately returned to the mode of ideation. By generating the outputs, the participants were able to generate a greater number of ideas, for instance, P03 in the CPS group stated—*“I was doodling without much thought and started to visualize it when doodling”*. This was followed by transition from preparation to preparation, which was 57% for the CPS group and 40% for the SPS group. Consequently, participants in the CPS group transitioned rapidly from the preparation-to-preparation stage to gather relevant information before moving on to the ideation stage. This is an important step as it is said that preparation requires 99% of perspiration which leads to 1% of inspiration [62]. For the CPS group, the transition from illumination to ideation was 65%, whereas there were no such instances in the SPS group. The reason for this is that the tasks in the SPS group did not require illumination activities, whereas in the CPS group, when the idea suddenly emerged, participants expected it to be developed further. For example, P06 in the CPS group reported—*“the idea that came instantly was the biryani competition”* (illumination) and then immediately reported—*“This was funny idea and I thought to develop it further”* (ideation).

More events of transition from production to preparation (82% vs. 12%), preparation to the production (50% vs. 19%), others to preparation (49% vs. 33%), illumination to preparation (100% vs. 5%), refinement to preparation (50% vs. 22%) happened for the SPS group than the CPS group, respectively. As part of the SPS group, it was necessary to examine sample sheets (preparation) to produce appropriate outputs. The results indicate that the preparation stage was most likely to occur for the SPS and was at the center of most of the transitions. It is likely that this result is due to the nature of the tasks where the participants spent most of their time collecting information and setting up the problem.

### SVM classification of stages based on facial AU combinations

For selecting the best separation between classes, the highest possible accuracy scores for each stage were considered. Among all the stages, only the target class block was classified with the highest accuracy scores (above 95%). The remaining stages were classified with moderate accuracy scores. The results are presented in Table 5.

**Table 5:** Accuracy scores of various stages for 7 emotions for the SPS and CPS groups.

Emotions	AU Combination	Accuracy scores in classification of stages											
		Preparation_CPS	Preparation_SPS	Ideation_CPS	Ideation_SPS	Evaluation_CPS	Evaluation_SPS	Refinement_CPS	Refinement_SPS	Production_CPS	Production_SPS	Block_CPS	Block_SPS
Anger	4+5+7+23	66.50%	59.91%	54.72%	61.01%	58.71%	59.94%	52.70%	49.39%	60.38%	62.45%	96.04%	97.26%
Happiness	6+12	67.65%	64.73%	73.81%	70.19%	72.25%	74.09%	85.81%	83.49%	74.35%	74.09%	45.70%	49.86%
Fear	1+4+5	68.97%	66.52%	56.90%	76.05%	60.74%	67.83%	49.49%	53.18%	60.55%	65.71%	94.67%	97.26%
Pride	6+7+12	63.03%	61.70%	55.27%	53.24%	64.63%	68.08%	81.58%	82.13%	70.29%	71.31%	43.98%	46.87%
Sadness	1+4+15	64.52%	61.01%	53.27%	63.69%	57.36%	60.45%	42.90%	43.70%	57.30%	58.67%	95.01%	95.95%
Disgust	4+7+9+10+17+20	56.43%	50.00%	39.45%	40.63%	45.17%	48.81%	33.95%	36.80%	47.88%	49.67%	96.90%	95.57%
Stress	1+6+12+15	58.08%	60.60%	43.09%	56.56%	59.72%	68.58%	77.19%	81.19%	60.71%	71.83%	57.73%	55.98%

#### Preparation stage and associated emotions

The stage preparation observed moderate accuracy scores for the ambivalent emotions, happiness (67.65% and 64.73%), and fear (68.95% and 66.52%) during the CPS and SPS process respectively. Positive emotions at the beginning of the task led some of the participants to approach the problem with high levels of motivation. For example, P01 (CPS) during task A reported—*“this question was very interesting and I was just staring at them to understand the question”* and P15 (CPS) reported,—*“I was totally excited so immediately I started counting the shapes*.*”* During task B, P21 (CPS) reported—*“I like literature and writing and I can use my knowledge so this was really exciting*,*”* and P03 (CPS) reported—*“this question was interesting as well as tough*.*”* During task C, P16 (CPS) reported—*“this task was full of creativity and fun*,*”* and P17 (CPS) reported—*“I thought why not first solve the 3rd task as it looks very interesting”*. For some of the participants, preparation was accompanied by a sense of apprehension after appraisal of the task about failing to complete the task. For example, during task A, P27 (CPS) reported—*“I was worried because I was losing on a lot of time in searching for the pen to write”*; P10 (CPS) reported—*“I was a bit skeptical and stressed when I read this question*.*”* Nevertheless, some participants also experienced negative emotions when attempting to complete the tasks on time. For example, during task A, P06 (CPS) reported—*“but these shapes were complex and I got scared to finish it on time*.*”* During task B, P13 (CPS) reported—*“basically this was a political question and I realized that this task was going to take a lot of time*.*”* During task C, P21 (CPS) reported—*“I was also thinking about how much time I have left for this task*. Participants in the SPS reported negative feelings in response to the focus and attention demanded by the tasks. For example, P15 (SPS) during task A reported—*“this one demanded focus and attention and I had to be careful in converting the shapes”*; P31 (SPS) during task B reported—*“so I was also calculating the values in my head after looking at these graphs*, *that’s why it was taking time for me to see and then write the answers”*; P17 (SPS) during the task C reported—*“this kind of task is like want you to be very attentive because you will have to measure everything and make them*.*”* Alternatively, the other perspective could be perhaps due to the nature of the tasks in the SPS group that were not exploratory and did not demand high divergent thinking abilities. For example, P06 (SPS) reported during task A—*“there was nothing much to think about this task”*; P31 (SPS) reported—*“actually this was a complicated problem due to the shapes and you can’t come up with ideas here*.*”*

#### Ideation stage and associated emotions

The ideation stage also involved ambivalent emotions. These observations are in accordance with previous findings [8]. Moderate accuracy scores were observed for happiness (73.81% and 70.19%) for the CPS and SPS groups, respectively. For fear, accuracy scores were significantly higher (76.05%) for the SPS group than for the CPS group (56.90%). Perhaps the gap between ideation and implementation of those ideas during the SPS process seemed farfetched due to the nature of the tasks that resulted in such a difference. Some of the participants dedicated their cognitive efforts on making richer associations with positive emotions, thereby facilitating flexibility. For example, P27 (CPS) during task A reported—*“so at first glance I thought that it was something like Picasso’s work and I was like very happy that I have direction now”*; P02 (CPS) during task A reported—*“it looked like a Dali painting somehow from which I got inspired*.*”* During task B, P15 (CPS) reported—*“I felt really happy that I came up with this idea of one nation one flag*.*”* While during task C, P06 (CPS) reported—*“I was thinking of making something similar to the Eiffel tower*.*”* Perhaps, for some of the participants, not being able to transform pre-inventive structures into a concrete mental representation or idea caused fear during ideation. For example, during task B, P33 (CPS) reported—*“I was scared if I will be able to complete this vague idea of UN ka intervention with Pakistan*.*”* In the SPS group, negative affects encouraged participants to develop self-efficacy necessary to reach the right solution, resulting in positive affect. The participant initially struggled with converting a few shapes in Task A, but later came up with an idea for transforming the shapes. For example, P02 (SPS) stated—*“I was not sure here but then I came across a rough idea of converting these arcs into semi triangle that made me feel lighter”*.

#### Block stage and associated emotions

Block is the only stage that observed very high accuracy scores for negative emotions—anger (96.04% and 97.26%), fear (94.67% and 97.26%), sadness (95.01% and 95.95%), and disgust (96.90% and 95.57%) respectively for the CPS and SPS groups. This makes sense as there are several factors that can be attributed to an impasse, for example, creative fixation, fear of failure, or lack of faith in oneself [63]. For example, during the task A, P1 (CPS) reported—*“this was a sad moment because I couldn’t think of anything at this moment”*; P2 (CPS) reported—*“I was not able to come up with an idea right away which pissed me off”*; P11 (CPS) reported—*“It was very irritating that why I am not able to move forward”*. During the task C, P19 (CPS) reported—*“this paper was frustrating me because there was nothing I can do with this”*. It is interesting to note, despite this, that some of the participants learned to accept these negative emotions, helping them to persist and complete their tasks, while others switched to an alternative activity.

Several participants in the SPS group were stuck on task A’s shapes, specifically the third element with two semi-circular shapes and the eleventh element with a star shape. For example, participant P01 (SPS) during task A reported—*“at this point I was confused and irritated about converting this circle or not”*; P02 (SPS) reported—*“then at this figure the confusion happened and I was fearing that I could make this one wrong”*; P30 (SPS) reported—*“this was not triangle not circle both which was frustrating to decode”*. In addition, some participants had difficulty rolling an 81-cm paper in Task C and encountered an impasse. For example, P04 (SPS) reported—*“First I was not satisfied and I did not understand what I have to do to make it 81 cms”*; P31 (SPS) reported—*“even after trying so hard it was not 81 cms which was irritating”;* P22 (SPS) reported—*“I was like there was no option to make the length bigger which was very sad”*. Participants in both the groups either abandoned the tasks immediately and switched to the other task or worked on the task for some time.

#### Evaluation stage and associated emotions

The evaluation stage observed moderate accuracy scores for ambivalent emotions, happiness (72.25% and 74.09%), pride (64.63% and 68.08%), fear (60.74% and 67.83%) and stress (59.72% and 68.58%) respectively for the CPS and SPS groups. This state, which requires focused attention to determine whether the idea is weak, is an emotionally taxing process. For example, P19 (CPS) during task C reported—*“but then this was not standing which gave me a lot of stress*.*”* P05 (CPS), during task A reported—*“but something was not very right here and it was very disappointing*.*”* Similarly, in the SPS group, P01 (SPS) during task A reported—*“in between I was checking whether I am doing it correctly as I was little worried”*; P11 (SPS) reported—*“I was having a lot of problem and stress in drawing this circle on this line”*. For task C, most of the participants utilized their cognitive efforts on measuring the length of the rolled sheet. For example, P13 (SPS) reported—*“mine was coming a bit long which was mind boggling”*; P27 (SPS) reported—*“when I realized this one to be 10 cms short I was like shit*…*”*

It is interesting to note that some participants appraised their results positively which perhaps resulted in experiencing happiness. For example, P03 (CPS), during task A, reported—*“I just looked at this thing and it came out to be nice*.*”* P22 (CPS) during task C, reported—*“here*, *when it stood na*, *I was very happy”*; P06 (CPS) reported—*“here I was happy that this structure could stand”*. Our findings are consistent with previous research that showed designers experience high levels of arousal and positive emotions during the verification stage [64].

#### Refinement stage and associated emotions

For the state of refinement, the CPS and SPS groups scored higher accuracy scores for ambivalent emotions—happiness (85.81% and 83.49%), pride (81.58% and 82.13%), and stress (77.19% and 81.19%) respectively. During this stage, additional layers of work, refinements, and finishing touches were added. Some participants were successful in resolving the issues and thus experienced happiness. For example, P01 (CPS) during task A reported—*“I felt extremely happy when I made more flames to provide a completeness in this photograph*.*”* Nonetheless, there were some participants who struggled to resolve the issues, which resulted in additional time and stress. For example, P14 (CPS) during task C reported—*“even after spreading these legs*, *my idea did not work that tensed me*.*”* P19 (SPS), during task A, reported—*“I shaded the rest of the shapes to be free from tension”;* P18 (SPS) reported—*“I was having problem in redrawing this circle again and again*.*”*

#### Production stage and associated emotions

This stage observed moderate accuracy scores for ambivalent emotions—happiness (74.35% and 74.09%), fear (60.55% and 65.71%), pride (70.29% and 71.31%), and stress (60.71% and 71.83%) for both the CPS and SPS groups respectively. At this stage, all the tasks required the participants to produce tangible outcomes to implement an idea they had already conceptualized. Perhaps, those who were successful in transforming their ideas into successful outcomes felt happiness and pride. For example, during task A, P17 (CPS) reported—*“once I had this face idea*, *I was successful in making it here*.*”* During task C, P02 (CPS) reported—*“I was enjoying making firm base out of this newspaper sheet*.*”* During task A, participant P18 (SPS) reported—*“I kept drawing like this because I was enjoying it*.*”* During task C, P22 (SPS) reported—*“I folded the sheet into exactly three parts and woaahhhh*!!*”*. Perhaps, some of the participants may have realized that they could not produce a quality outcome and, as a result, might have experienced fear and stress. The realization of this ongoing process of production differs from the process of evaluation where the produced outcome or an idea is verified. It becomes difficult to capture the evaluation of the ongoing production process during the retrospective think-aloud protocol.

## General discussion and conclusion

For a more comprehensive understanding of creativity, it is necessary to capture creativity act in its totality and the underlying emotional mechanisms. This work presents a novel method of capturing in-the-moment emotional experiences of the individuals during the creative process using the facial expressions data and a headgear setup. Previous studies have leveraged self-report scales and diary studies in examining the emotion influences on the creative work. This study is first to capture the emotional states associated with each stage during the CPS process using real-time facial action unit combinations and support vector machine classification. Moreover, this work also compared the CPS and SPS processes using the protocol analysis and Markov chains.

In order to answer the first research question (RQ1—what stages of the CPS and SPS require the greatest amount of effort from the individuals?), protocol analysis and Markov’s analysis results revealed that the participants in the CPS group spent most of their cognitive effort during the stages of ‘ideation,’ ‘illumination,’ and ‘refinement,’ whereas the participants in the SPS group spent most of their cognitive effort during preparation and production. Perhaps, this difference is attributable the tasks assigned, as the tasks in the CPS group offered more opportunities for divergent thinking. In the ‘ideation’ process, participants spent most of their time deriving pre-inventive structures, although some participants were more successful than others in generating pre-inventive structures quickly and solving the tasks on time. Different components, such as task motivation, and skillsets impacted the quality of output produced for a specific task as not all tasks were attended with equal efforts. The same is true for the other stages. For example, most participants showed more interest in solving tasks A and C than task B for the CPS group. Although all these tasks encouraged divergent thinking, drawing and structure-making tasks were considered more playful and enjoyable than the written tasks. Task A and Task B were given priority over task C for the SPS group. This requirement arose from the requirement for greater focus and attention in reading the instructions and then developing the prototype. Furthermore, all the tasks in the SPS group involved high attentional demands, which meant some participants were able to focus more and produce better results. As a result of this study, one of the approaches proposed by [2] to differentiate between the CPS and SPS is supported: these two processes entail the same stages, but the variation is in the level of execution at each stage. These results provide the first empirical evidence in understanding the difference between the CPS and SPS process at a micro-level and lend support to studies suggesting that the creative process must allow higher degrees of divergent and convergent thinking [6]. There were also differences in transition state diagrams between the CPS and SPS processes. The CPS group experienced more transitions from ‘preparation’ → ‘preparation,’ ‘illumination’ → ‘ideation’ and ‘production’ → ‘ideation.’ The SPS group, on the other hand, experienced more transitions from ‘production’ → ‘preparation,’ ‘refinement’ → ‘preparation,’ ‘illumination’ → ‘preparation,’ ‘others’ → ‘preparation’ and from ‘preparation’ → ‘production.’

To address the second research question (RQ2—What kinds of emotions occur at different stages of the creative process?), the exploratory part of this study investigated the in-the-moment experience of emotions during the CPS processes by fulfilling the research gap posited in the literature regarding the use of automatic facial expression analysis in researching the role of emotions in creativity. Six stages were classified using SVM based on seven facial expressions (or facial AU combinations). Although ambivalent emotions were majorly present during most of the creative stages, considering the results, specific emotions were found to be present for different stages where the negative and positive emotions have a predominance presence. Happiness and pride were found to be predominant during the stages of evaluation, refinement, and production whereas negative emotions were predominant during the stage of block. These results are unique and corroborate with the previous findings. During the evaluation and refinement process, one may see a silver lining that induces happiness whereas, during a creative block, the idea becomes fixated, and the emotions become predominantly negative.

The findings of this study are consistent with theories relating to the role of ambivalent emotions in creative work [18, 22, 25]. The appraisal of the tasks and the appraisal of one’s performance during the completion of the tasks caused most participants to experience both positive and negative emotions at different time intervals. Furthermore, the time pressure to finish the three tasks on time allowed some participants to stay motivated and experience ambivalent emotions to be more creative, but for others, this time pressure acted as a guillotine to their creative performance. On the contrary, the stage of ‘block’ was highly dominated by negative emotions, and high accuracy scores were observed for anger, fear, sadness, and disgust. This makes sense as the previous research has indicated that a diverse set of negative thoughts is encountered during the resolution of this state, e.g., loss of focus, fear of failure, worry, depression, etc [53]. Interestingly, we did not observe much difference in the emotional profiles for each stage between the CPS and SPS processes, even though CPS and SPS have different distributions of stages.

### Limitations of the study

Unlike any other study, this study has its limitations. We did not postulate a dichotomy between the CPS and SPS processes nor examine the different categories of stages between the two processes. Segmenting and coding different categories of stages for the CPS and SPS processes might pose a construct validity issue since the literature fails to distinguish stage-based differences for the CPS and SPS processes. In addition, conducting a statistical test becomes difficult based on the difference in categories of stages between the CPS and SPS stages. Another limitation of the study is its lack of ecological validity. The creative process in the real physical world is influenced and mediated by many factors, and thus, the laboratory-based study may yield a different result from those in the natural situation [65]. As of now, the prospect of recording and analyzing moment-to-moment facial expressions in a real-life situation seems distant at this moment. Additionally, this research was constrained by the retrospective protocol analysis, which lowered the accuracy of the in-the-moment CPS and SPS process analyses. Nevertheless, concurrent verbal utterances could have introduced bias in the data due to redundant facial muscles movements. Furthermore, the small sample size didn’t permit us to account for the differences between the creative performances of the high and low scorers.

### Implications of this research

Despite these limitations, this research can have significant practical implementations in creativity research. Novel methods and tools could be developed to train individuals to recognize and regulate their emotions during the creative process to create a deeper understanding of what emotions to expect at each stage and implement effective strategies to manage those emotions. The process of ideation could, for instance, be enhanced by inducing positive emotions and allowing flexibility. Similarly, creative individuals can also be trained to regulate their negative emotions during the creative block to stay motivated and find ways to solve the problem. Few studies have developed personalized gaming experiences based on different emotions. E.g., the game alters its difficulty level by sensing negative emotions in gamers through recognizing facial expressions in real-time to help them stay motivated throughout the gameplay [66, 67]. Consequently, the results of this study may serve as a baseline for developing creative support systems for various professionals. For instance, an individual could receive personalized visual cues or affective cues in real-time based on the changes in their facial expression captured by their personal device to enhance their creative performance. This claim may be considered immature at this point due to the limitations of current technological advances, as well as laws and policies about the monitoring of sensitive facial information. Furthermore, designing such affective solutions must demonstrate excellent reliability and validity to be generalized.

### Future work

There is a need for further research regarding creativity and facial expressions. Replication of our study with a larger sample size among various ethnic groups may demonstrate the similarities and differences with our findings. Future work could include longitudinal studies that capture facial expressions at different stages of the creative process to clarify the relationship between creativity and emotions. Moreover, the benefits of such studies could also improve ecological validity by determining the causal connection. A further step could be to triangulate this study by utilizing other technological interventions, e.g., EEG and biofeedback sensors. In addition, different automatic facial expression recognition algorithms could be used to cross-validate the accuracy scores obtained in this study.

## Supporting information

S1 Dataset (TXT)
