# Correction: Creative problem solving and facial expressions: A stage based comparison

**DOI:** 10.1371/journal.pone.0304235

**Published:** 2024-05-17

**Authors:** Mritunjay Kumar, Satyaki Roy, Braj Bhushan, Ahmed Sameer

The images for Figs 6 and 7 are incorrectly switched. The image that appears as Fig 6 should be Fig 7, and the image that appears as Fig 7 should be Fig 6. Also, there is an error in the captions of the figures. Please see the correct Figs 6 and 7 and its captions here.

**Fig 6:**
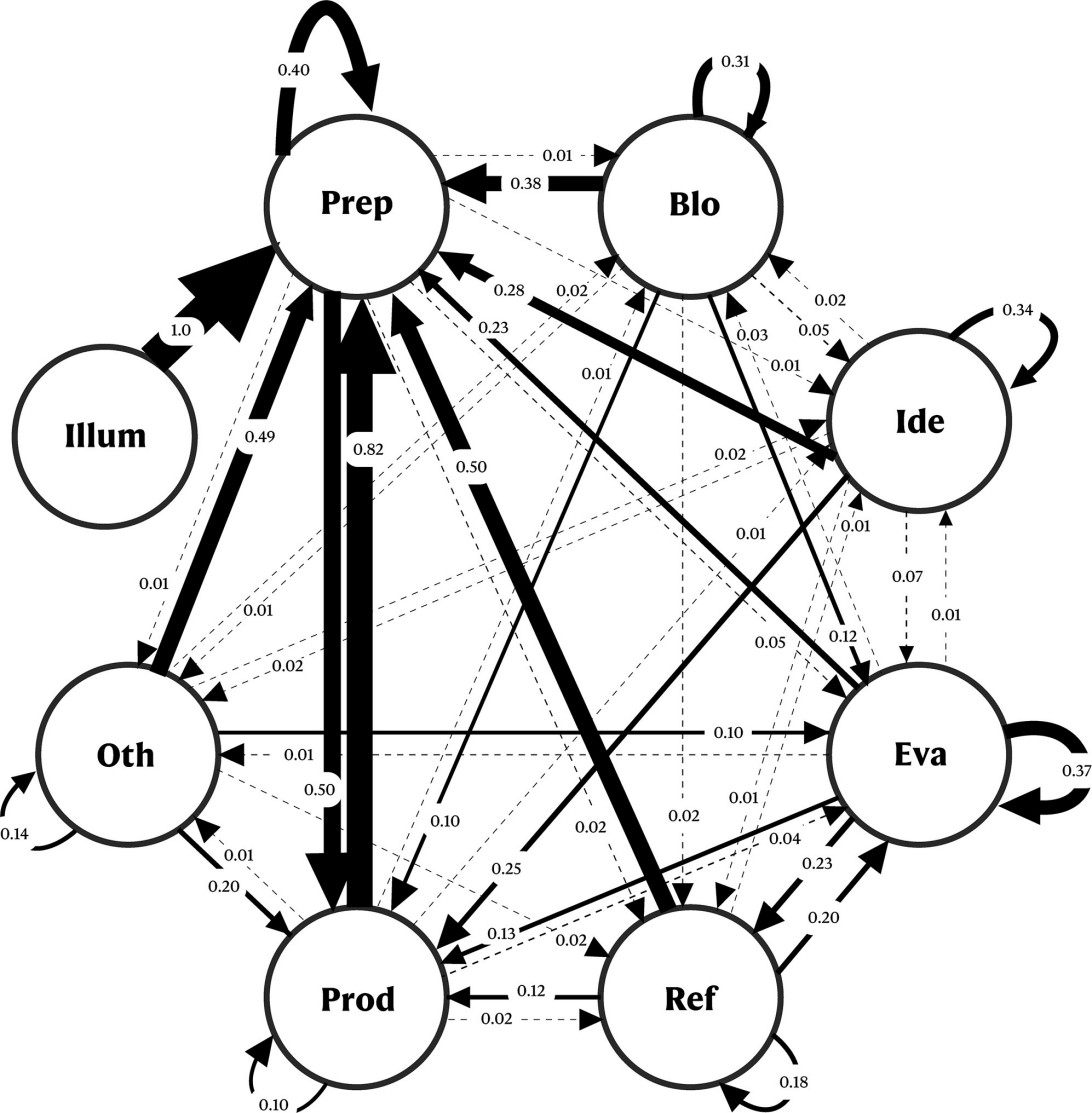
Diagram of transitional states for the various stages of the CPS group. Blo = Block; Ide = Ideation; Eva = Evaluation; Ref = Refinement; Prod = Production; Oth = Others; Illum = Illumination.

**Fig 7:**
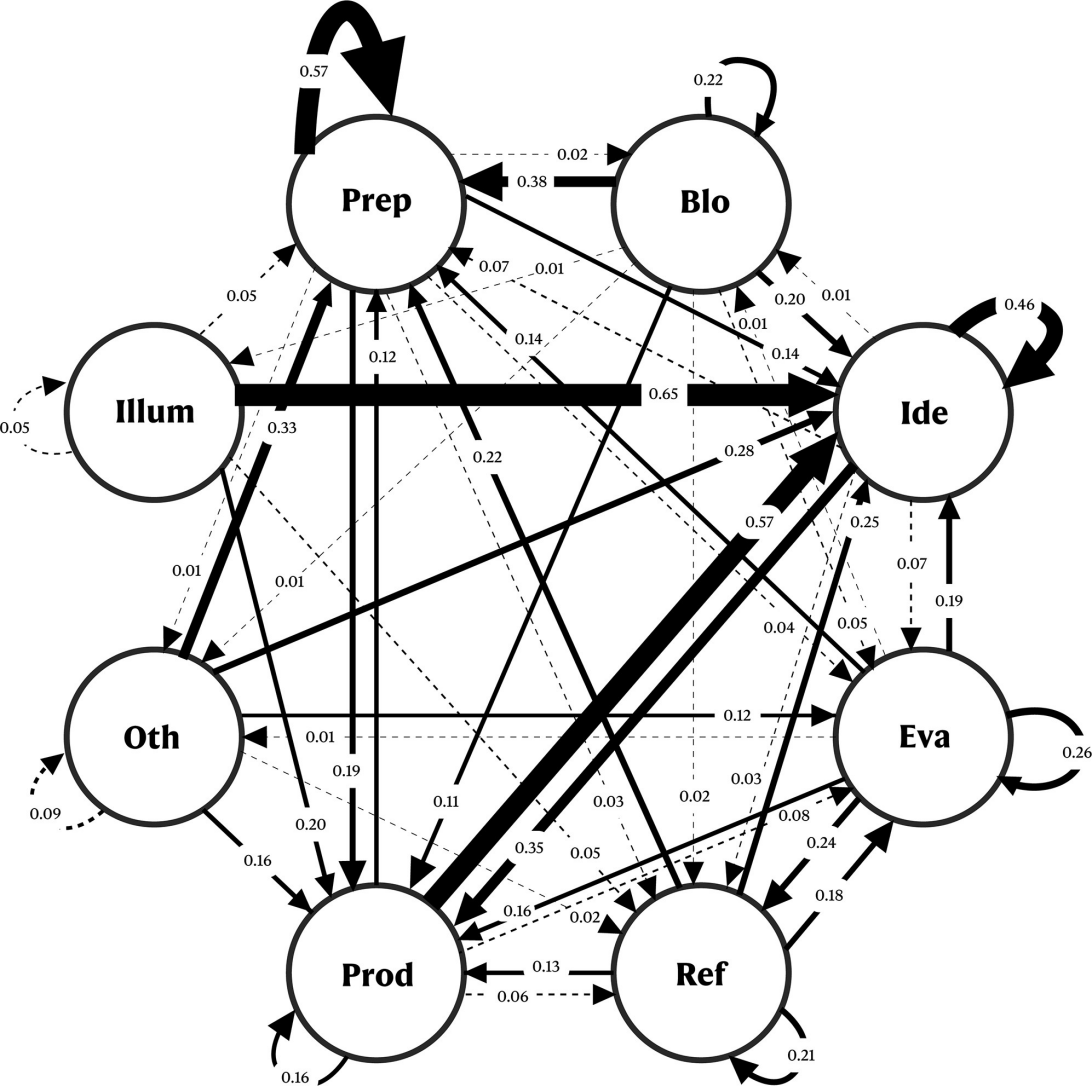
Diagram of transitional states for the various stages of the SPS group. Blo = Block; Ide = Ideation; Eva = Evaluation; Ref = Refinement; Prod = Production; Oth = Others; Illum = Illumination.

Moreover, in the Facial expression analysis subdivision of Data analysis, the word “Noldus” is misspelled in the second sentence of the second paragraph. The correct sentence is: Based on comparing the performance of automatic facial expression recognition tools, OpenFace has been shown to be superior to the high-paid commercial softwares, e.g., Noldus FaceReader, and Affectiva in detecting automatic facials.
